# Arterial Stiffness and Indices of Left Ventricular Diastolic Dysfunction in Patients with Embolic Stroke of Undetermined Etiology

**DOI:** 10.1155/2019/9636197

**Published:** 2019-09-12

**Authors:** Paulina Gąsiorek, Agata Sakowicz, Maciej Banach, Stephan von Haehling, Agata Bielecka-Dabrowa

**Affiliations:** ^1^Department of Neurology and Ischemic Strokes, Medical University of Lodz, Zeromskiego 113, 90-549 Lodz, Poland; ^2^Department of Medical Biotechnology, Medical University of Lodz, Poland; ^3^Department of Hypertension, Chair of Nephrology and Hypertension, Medical University of Lodz, Poland; ^4^Department of Cardiology and Congenital Diseases of Adults, Polish Mother's Memorial Hospital Research Institute (PMMHRI), Rzgowska 281/289, 93-338 Lodz, Poland; ^5^Department of Cardiology and Pneumology, University of Göttingen Medical Center, Göttingen, Germany; ^6^German Center for Cardiovascular Research (DZHK), Partner Site Göttingen, Germany

## Abstract

**Purpose:**

The study is aimed at identifying echocardiographic and circulating biomarkers as well as hemodynamic indices of embolic stroke of undetermined etiology (ESUS) in patients aged <65.

**Methods:**

We prospectively investigated 520 patients with confirmed ischemic stroke and selected those 65 patients who were diagnosed with ESUS (age 54 (47-58) years, 42% male). An additional 36 without stroke but with a similar risk profile were included as a control group (age 53 (47-58) years, 61% male). All patients underwent echocardiography, noninvasive assessment of hemodynamic parameters using a SphygmoCor tonometer (AtCor Med., Australia), and measurements of selected biomarkers.

**Results:**

ESUS patients and controls were well matched for baseline characteristics including blood pressure and left ventricular ejection fraction (LVEF). Compared to controls, patients with ESUS had lower mean early diastolic (*E*′) and systolic (*S*′) mitral annular velocities and a higher ratio of the peak velocity of early diastolic transmitral flow to the peak velocity of early diastolic mitral annular motion (all *p* < 0.01). The peak velocity flow in the late diastole (A wave) value and LV mass indexed to the body surface area (LVMI) (g/m^2^) were higher in the ESUS group than in the control group (both *p* < 0.01). The isovolumetric relaxation time (IVRT) was longer and the mean left atrial volume index (LAVI) was higher in ESUS patients compared to the control group. Parameters of arterial stiffness such as augmentation pressure, augmentation index, and augmentation index adjusted to a heart rate of 75 bpm (AIx75) were higher in ESUS patients compared to controls (*p* < 0.05). Patients in the ESUS group had higher levels of asymmetric dimethylarginine, interleukin 6, and N-terminal probrain natriuretic peptide (NT-proBNP, all *p* < 0.05) than those in the control group. In multivariate analysis, the following factors were significantly associated with the presence of ESUS: AIx75 (odds ratio (OR) 1.095, 95% confidence interval (CI) 1.004-1.194; *p* = 0.04), IVRT (OR 1.045, 95% CI: 1.009-1.082; *p* = 0.014), LAVI (OR 1.3, 95% CI: 1.099-1.537; *p* = 0.002), and NT-proBNP (OR 1.003, 95% CI: 1.001-1.005; *p* = 0.005).

**Conclusions:**

Increased arterial stiffness and indices of diastolic dysfunction as well as a higher NT-proBNP level are significantly associated with ESUS. These parameters require further scrutiny over time to understand their impact on the development of symptomatic heart failure. The ClinicalTrials.gov identifier is NCT03377465.

## 1. Introduction

More than one million inhabitants of Europe suffer from stroke yearly, and ischemic stroke accounts for approximately 80% of all cases. Despite the reduction in stroke mortality, the absolute number of people with stroke-related death has increased greatly in the past two decades [1, 2]. Identification of the etiology of stroke is necessary to prepare an adequate prevention strategy [3]. The term embolic stroke of undetermined etiology (ESUS) was introduced by the Cryptogenic Stroke (CS)/ESUS International Working Group in 2014 [4]. ESUS refers to a nonlacunar infarct, which means a subcortical infarct ≤ 1.5 cm on computed tomography or ≤2.0 cm on magnetic resonance imaging in the absence of the following: cardioembolic sources such as permanent or paroxysmal atrial fibrillation (AF) or atrial flutter, intracardiac thrombus or tumors, prosthetic cardiac valve, mitral stenosis, myocardial infarction within the past 4 weeks, left ventricular ejection fraction < 30%, valvular vegetations, or infective endocarditis as well as extracranial or intracranial atherosclerosis causing >50% luminal stenosis in the artery supplying the ischemic region and other specific causes of stroke (e.g., dissection, arteritis, migraine/vasospasm, and drug misuse) [4, 5]. Approximately one-fourth of all strokes are ESUS. Identification of the prognostic factors is necessary in order to optimize the preventive strategy [6]. The presence of ESUS strokes indicates that the conventional risk factors cannot fully account for the pathogenesis of stroke. The characteristics and predictors of ESUS stroke in patients with heart failure without significant LVEF reduction and without AF are unknown [7]. A growing number of studies have demonstrated the association between parameters of arterial stiffness and stroke [8]. Endothelial dysfunction assessed by an increased level of asymmetric dimethylarginine (ADMA) may affect the inflammatory state in patients with ESUS [9]. It is very important to detect useful biomarkers of the risk of ESUS for appropriate intervention. The aim of this study was to identify echocardiographic and circulating biomarkers as well as hemodynamic indices of embolic stroke of undetermined etiology (ESUS) in patients aged <65.

## 2. Methods

### 2.1. Study Population

We prospectively investigated 520 patients with confirmed ischemic stroke hospitalized in the Department of Neurology and Ischemic Strokes, Medical University of Lodz [10]. We enrolled patients (males, females; age median 54 (interquartile range, IQR 47-58) years) with ESUS and 36 to the control group (median 53 age 47-58 years, 61% male) from the Department of Hypertension, Medical University of Lodz. All patients underwent neuroimaging examination, arterial ultrasound examination, electrocardiogram (ECG) monitoring, echocardiography, and noninvasive assessment of hemodynamic parameters using a SphygmoCor tonometer [9, 11]. Other measurements obtained included the levels of selected biochemical biomarkers.

We define ESUS as nonlacunar stroke with no major-risk cardioembolic source of embolism and with the absence of extracranial or intracranial atherosclerosis causing 50% stenosis in the arteries supplying the area of ischemia and with no other specific causes of stroke [4, 5].

The exclusion criteria were as follows: unstable hypertension, atrial fibrillation, hyperthyroidism, pregnancy and breastfeeding, dialysis, cancer, autoimmunologic disease, reception of cytostatic, immunosuppression drugs, glucocorticosteroids, antiretroviral drugs, transplant and treatment with hematogenous preparation during the last 6 months, active infection, alcoholism, addiction to medicines, infection with hepatitis B virus (HBV), hepatitis C virus (HCV), human immunodeficiency virus (HIV), surgical intervention or serious injury during the last 1 month, vaccination during the last 3 months, and incapable of giving agreement.

All enrolled patients underwent blinded adjudication by cardiologists experienced in adjudication. Detailed clinical, imaging, and biomarker data were collected at the time of enrollment, and echocardiogram and SphygmoCor analyses were performed and interpreted by doctors blinded to biomarker analysis. Central systolic and diastolic pressures were measured using a sphygmomanometer and peripheral pressures using a stethoscope.

All methods in this study were performed in accordance with the guidelines and regulations approved by the Bioethics Commission of the Medical University of Lodz, and approval from this commission (no. RNN/272/16/KE) was obtained. Written informed consent was obtained from all the patients. The study was registered at ClinicalTrials.gov—identifier number: NCT03377465 (Biomarkers, Hemodynamic and Echocardiographic Predictors of Ischemic Strokes and Their Influence on the Course and Prognosis) [9, 12].

### 2.2. Echocardiography

All patients were examined following a standardized protocol using an ALOKA Alpha 10 Premier (Tokyo, Japan) with a 3–11 MHz probe after inclusion. Quantitative echocardiography was used following current guidelines [13]. Left ventricular volumes and ejection fraction (EF) were determined by biplane Simpson's method [14]. The left ventricular mass was calculated using the Devereux formula. The early (*E*) and atrial filling (*A*) peak velocities, *E*/*A* ratio, deceleration time of early filling, and isovolumic relaxation time were measured from the transmitral flow. Peak systolic (*S*′), early diastolic (*E*′), and late diastolic (*A*′) mitral annular myocardial velocities of the left ventricle septal and lateral walls were recorded from the apical 4-chamber view with pulsed wave tissue Doppler, and results were averaged. The *E*/*E*′ was calculated as an index of LV filling pressure [13].

### 2.3. Laboratory Tests and Biomarkers

Blood samples for laboratory tests were collected from patients assigned to either group in a hospital setting, thus minimizing the risk of infection in both the subject and the person collecting the sample. Laboratory tests were performed in fasting subjects in a laboratory of WAM Hospital, following a minimum 12-hour period after the last meal. At the initial time point of the study, 19.5 mL of blood was collected with a vacuum blood collection system from the basilic vein into 8.5 mL, 5 mL, and 4 mL clot activator plastic Vacutainer tubes and into 2 mL Vacutainer tubes containing ethylenediaminetetraacetic acid (EDTA), for routine laboratory tests. The blood samples to perform biomarker analysis were taken on the 7th day after stroke. Enzyme-linked immunosorbent assay (ELISA) tests were conducted for quantitative determination of N-terminal probrain natriuretic peptide (NT-proBNP) (Cloud-Clone-Corp., China), interleukin 6 (IL-6) (Gen-Probe, France), and asymmetric dimethylarginine (ADMA) (Immundiagnostik, Bensheim) in human serum [9].

### 2.4. Holter ECG

72 h Holter ECG was recorded in a 2-channel, 5-electrode paradigm with a GE SEER Light Ambulatory Recorder and analyzed with the GE Marquette MARS Holter System (GE Medical Systems, Milwaukee, WI 53223, USA) [15].

### 2.5. Noninvasive Assessment of Hemodynamic Parameters Using the SphygmoCor System

#### 2.5.1. Central Blood Pressure

The central (ascending aortic) pressure waveform was derived by radial applanation tonometry 7 days after stroke and application of a generalized transfer function to the radial pressure waveform using a commercial device (SphygmoCor 9.0; AtCor Medical, Sydney, Australia) [16]. Central augmented pressure (AP) was calculated as the difference between the first and second systolic peaks on the central pressure waveform. The aortic augmentation index (AIx), a composite marker of systemic arterial stiffness and left ventricular afterload, was calculated by AP as a percentage of the total pressure waveform height. Our aim was to achieve high-quality waveforms indicated by a pulse height of >100, with a pulse length and diastolic variation ≤ 5.

#### 2.5.2. Arterial Stiffness

Central arterial stiffness was assessed by aortic pulse wave velocity (PWV) using electrocardiogram-gated sequential tonometry at the carotid and radial sites (SphygmoCor 9.0; AtCor Medical, Sydney, Australia) [16]. The path length was calculated by subtracting the distance between the sternal notch and carotid recording site from the distance between the sternal notch and the radial site. The aortic systolic pressure, aortic diastolic pressure, aortic pulse pressure, mean aortic pressure [17], pulse wave velocity (PWV), augmentation pressure (AP), and augmentation index (AIx) were obtained 7 days after stroke. AIx and AP were derived by pulse wave analysis (PWA) [18]. AP is the maximum systolic pressure minus pressure at the inflection point. PWV was calculated as the path length divided by transit time (meters/second). The average of measurements over a period of 11 s (9–10 cardiac cycles) was calculated after the exclusion of extreme values [18].

#### 2.5.3. The SphygmoCor Heart Rate Variability Assessment System

The SphygmoCor heart rate variability system is a sophisticated system for noninvasively assessing the autonomic nervous system (ANS) based on heart rate variability (HRV) analysis. HRV analysis is based on measuring variability in intervals between R waves (i.e., R-R intervals) [18].

### 2.6. Statistical Analysis

The STATISTICA 13.1 software package (StatSoft, Poland) was used for analysis. Results were considered significant if *p* < 0.05. The Shapiro-Wilk test was used to assess the normality of distribution. Data were presented as mean and standard deviation or median and interquartile range (25%-75%), depending on the data scale and distribution. To compare two groups, Student's *t*-test for continuous variables with normal distribution and with homogeneity of variance was used. For data with normal distribution but lacking homogeneity of variance, the Welsh test was conducted. The Mann-Whitney *U* test for nonnormally distributed variables was used.

The dichotomous data were analyzed by the chi-square test or chi-square with Yates correction. Variables significant in univariate analysis (significance level *p* < 0.05) were used for the construction of a multivariate logistic regression model; logistic regression was conducted among the patients from the ESUS group (*n* = 65) vs controls (*n* = 36). The quality of the models and the usefulness of the markers were evaluated using receiver operating characteristic (ROC) curves and tables of reclassification. For quantitative variables (continuous and discrete) to evaluate correlations between variables, Spearman's rank correlation coefficient was used.

## 3. Results

### 3.1. General Characteristics of Patients

There were no significant differences between groups in the body mass index (BMI), peripheral systolic and diastolic blood pressures, or additional diseases such as coronary artery disease (CAD), hypertension, and diabetes. Patients with ESUS more frequently were smokers (38% vs 13%; *p* = 0.02). In the group with ESUS, 23% of patients received ASA (acetylsalicylic acid) and statin, 28% beta blocker, 37% ACE (angiotensin-converting enzyme), and 20% CCB (calcium channel blocker) and 20% took a diuretic and 8% insulin. In the control group, 9% of patients received ASA, 17% statin, 12% beta blocker, 24% ACE, 9% CCB, and 15% diuretic and none of them took insulin before admission to the hospital. There were no differences between the stroke and control groups in the levels of low-density lipoprotein (LDL) cholesterol, total cholesterol, triglycerides, prothrombin time, or activated partial thromboplastin time. The level of high-density lipoprotein (HDL) cholesterol was significantly lower in the ESUS group than in controls (1.19 mmol/L (0.95-1.46) vs 1.37 (1.19-1.6); *p* = 0.02). Patients in the stroke group had higher levels of NT-proBNP pg/mL (391 (107-1249) vs 109 (46-236); *p* = 0.003), IL-6 pg/mL (2.6 (0.8-8.1) vs 0.7 (0.4-1.2); *p* = 0.002), and ADMA *μ*mol/L (0.44 (0.39-0.55) vs 0.36 (0.32-0.4); *p* = 0.0002) than the control group (18 congress abstract). Patients in the ESUS group had higher levels of glomerular filtration rate (GFR) mL/min/1.73 m^3^ (75 (64-89) vs 68 (62-78); *p* = 0.002) compared to the control group.

The basic characteristics of patients in groups are presented in Tables 1 and 2.

### 3.2. Findings on Echocardiography

There were no differences in aortic diastolic pressure (DP) and systolic pressure (SP) between ESUS and control groups. ESUS patients had a lower value of left ventricular ejection fraction (LVEF) than patients from the control group, but in both groups, the values were proper (60 (55-64) % vs 63 (60-66) %; *p* = 0.009). ESUS patients also had lower mean early diastolic (*E*′) (median 8.6 cm/s (7.1-10.3) vs 12.5 cm/s (9.6-14); *p* = 0.0008) and systolic (*S*′) mitral annular velocities (mean 7 ± 1 vs 8 ± 1 cm/s; *p* = 0.03) and a higher *E*/*E*′ ratio compared to the control group (median 7.6 (6.1-8.9) vs 6.0 (5.3-6.9), *p* = 0.0002). The peak velocity flow in the late diastole (A wave) value and LV mass indexed to the body surface area (LVMI) (g/m^2^) were higher in the ESUS group than in controls (80 ± 19 vs 64 ± 17 cm/s; *p* = 0.01 and 112 (90-125) vs 89 (77-101); *p* = 0.0004). Isovolumetric relaxation time (IVRT) was longer in ESUS patients compared to the control group (113 ± 23 vs 97 ± 30 ms; *p* = 0.001). The mean left atrial volume index (LAVI) was higher in the ESUS group (27 ± 11 vs 21 ± 5; p = 0.01). ESUS patients (25% of them) more frequently had nonhemodynamically significant liquid in the pericardium (*p* = 0.04) compared to the control group (6%) (11).

The evaluation of selected echocardiographic parameters in groups is presented in Table 3.

### 3.3. Noninvasive Assessment of Hemodynamic Parameters Using the SphygmoCor System

The parameters of arterial stiffness augmentation pressure (AP), augmentation index (AIx), and augmentation index adjusted to a heart rate of 75 bpm (AIx75) were higher in ESUS patients compared to controls (11 mmHg (7-18) vs 6 (3-13); *p* = 0.001, 27 ± 13 vs 22 ± 13%; *p* = 0.03, and 25 ± 11 vs 18 ± 12; *p* = 0.009, respectively). Aortic systolic pressure (SP) was higher in the ESUS group (125 ± 16 mmHg vs 116 ± 7; *p* = 0.01), and the heart rate variability (HRV) index was lower in the ESUS group compared to controls (6.6 (4.7-9) vs 8.7 (5.9-12); *p* = 0.006). The evaluation of hemodynamic parameters using the SphygmoCor system is presented in Table 4.

### 3.4. Multivariate Logistic Regression Analysis

The significantly associated parameters in the univariate logistic regression analysis presented in Table 5 were used in the multivariate regression analysis. In the multivariate analysis, the factors independently associated with the presence of ESUS were AIx75 (odds ratio (OR) 1.095, 95% CI 1.004-1.194; *p* = 0.04), IVRT (OR 1.045, 95% CI: 1.009-1.082; *p* = 0.014), LAVI (OR 1.3, 95% CI: 1.099-1.537; *p* = 0.002), and NT-proBNP (OR 1.003, 95% CI: 1.001-1.005; *p* = 0.005). This analysis is presented in Table 6.

**Figure 1:**
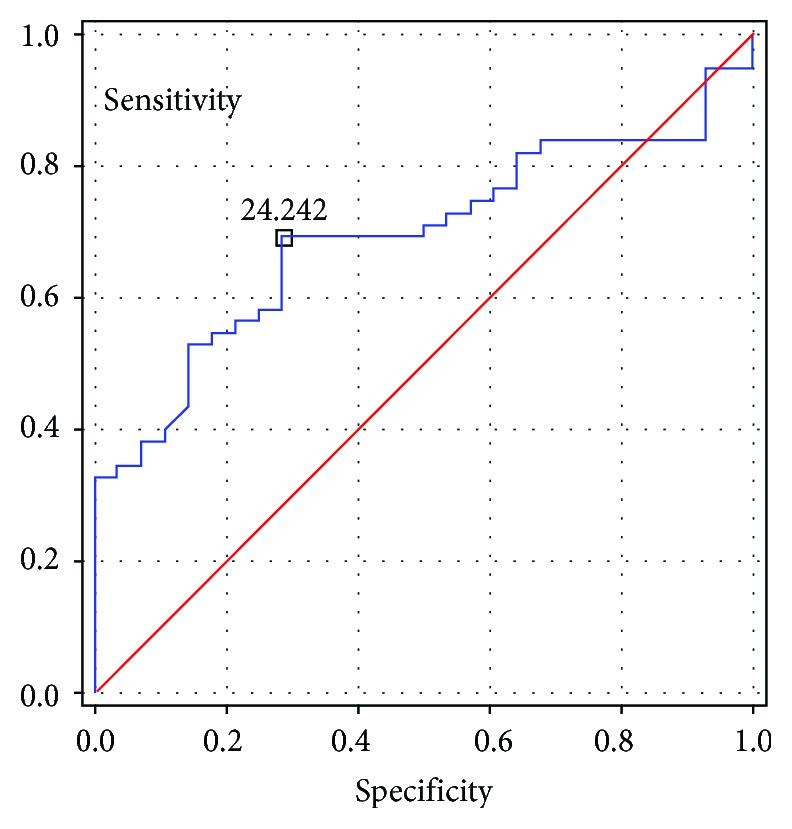
ROC chart for LAVI.

**Figure 2:**
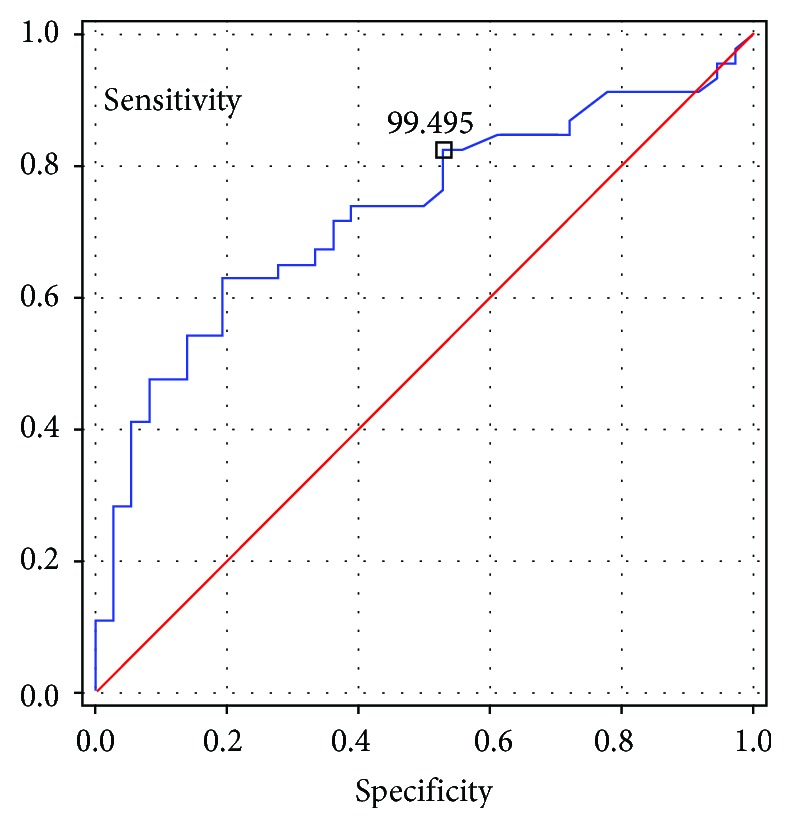
ROC chart for NT-proBNP.

The value of LAVI more than 24 mL/m^2^ and NT-proBNP values higher than 99.5 pg/mL were associated with the presence of ESUS. The ROC charts for LAVI and for NT-proBNP are presented accordingly in Figures 1 and 2 (11).

## 4. Conclusions

Increased arterial stiffness and indices of left ventricular diastolic dysfunction as well as a higher NT-proBNP level are associated independently with the presence of ESUS. Our results suggest that there may be a role for increased heart rhythm surveillance in patients with indices of diastolic heart failure to prevent stroke. Despite the lack of long-term randomized double-blind controlled therapeutic trials, there is high potential to reduce stroke prevalence through a significant reduction of arterial stiffness. Pharmacological interventions and lifestyle modification that can influence blood pressure, arterial function, or structure in either the short or long term are promising therapies reversing arterial stiffness, which can prevent ESUS strokes.

## 5. Discussion

### 5.1. Principle Findings

The results of this study revealed that ESUS patients had lower mean early diastolic (*E*′) and systolic (*S*′) mitral annular velocities and a higher *E*/*E*′ ratio as well as longer IVRT compared to the control group (11). Also, mean LAVI and the level of NT-proBNP were higher in the ESUS group, which suggests that despite the proper values of LVEF, the indices of diastolic heart failure could be a predictor of this type of stroke. The parameters of arterial stiffness—augmentation pressure (AP), augmentation index (AIx), and augmentation index adjusted to a heart rate of 75 bpm (AIx75)—were also higher in ESUS patients compared to controls. The parameters independently associated with the presence of ESUS were as follows: higher AIx75, longer IVRT, higher LAVI, and higher NT-proBNP levels. The cutoff points for LAVI and NT-proBNP indicating an increased risk of ESUS were lower in our study than those accepted in the standards for the diagnosis of heart failure (125 pg/mL compared to 99 pg/mL in our study) and LAVI (34 mL/m^2^ compared to 24 mL/m^2^ in our study). Increased arterial stiffness and indices of diastolic heart failure are associated independently with the occurrence of ESUS (11).

### 5.2. Indices of Diastolic LV and LA Dysfunctions and Risk of Stroke

Patients with heart failure with a reduced ejection fraction (HFrEF) are at risk from thromboembolic events originating from both the arterial and venous circulation, which is in part a result of Virchow's triad of risk factors for thrombus formation strictly connected with heart failure syndrome [19–21]. Kodiak et al. investigated the association between left ventricular diastolic dysfunction (LVDD) and stroke of different origins as well as cryptogenic stroke. Their results showed that the CHA2DS2-VASc score was higher in patients with LVDD [22]. In addition, LVDD compared to the CHA2DS2-VASc score was a stronger predictor of stroke in AF patients. In the study of Najafi-Dalui et al., LVDD was not associated with the CHA2DS2-VASc score in patients with nonhemorrhagic stroke and coexisting AF [22, 23]. Up to half of patients with heart failure have heart failure with preserved ejection fraction (HFpEF). The prognosis of HFpEF patients is considerably worse than that of patients with coronary artery disease, hypertension, AF, or diabetes in the same age range and gender distribution. Little is known about the incidence of stroke in HFpEF, particularly in the absence of AF. The Atrial Fibrillation Clopidogrel Trial with Irbesartan for Prevention of Vascular Events (ACTIVE trial) which included over 3400 patients with AF showed similar risks (hazard ratio of 1.01; 95% confidence interval, 0.78–1.31) of 4.3% and 4.4% per 100 person years for embolic events in noncoagulated patients with HFpEF and in HFrEF, respectively [24, 25]. Abdul-Rahim et al. revealed a similar risk of stroke in patients without AF with HFpEF (1.0% per year) and HFrEF (1.2% per year) and concluded that routinely collected clinical variables may help clinicians to identify patients with HFpEF, who may have sufficiently high risk of stroke although they do not have AF potentially to justify anticoagulation [7]. Cogswell et al. hypothesized a possible influence of silent paroxysmal AF on stroke risk in HFpEF patients, given that stroke risk in patients with HFpEF without AF and HFpEF with AF as well as AF only was similar [26, 27]. Based on the Atherosclerosis Risk in Communities (ARIC) Study with 1,527 participants, the authors concluded that undetected AF may be common in patients with HFpEF and not detecting this may lead to associated cerebral infarcts. Risk factors for having cerebral infarcts in the HFpEF/no AF group included left atrial enlargement [25]. Left atrium (LA) function comprises reservoir, conduit, and pump functions, which are dependent on left ventricular diastolic function. The left atrial volume index (LAVI), a biomarker of LA dysfunction reflecting the aggravation of diastolic LV function, is strongly associated with cardiovascular disease and outcomes [28]. In the study by Lee et al., the authors compared the LAVI values between ESUS patients with patent foramen ovale (PFO) and healthy subjects with PFO and found that the ESUS patients had larger LA volumes than controls regardless of the presence of PFO. What is interesting is that LA enlargement, but not the amount of shunting, was associated with cortical infarctions, which could imply recurrent embolic stroke [29, 30]. Also, Lee et al. suggested that LA dysfunction could be a marker of incident AF, atrial thrombi, and thromboembolic risks of AF [31]. CHA_2_DS_2_-VASc is the most widely accepted scoring system to assess stroke risk in AF patients, although still we do not have enough information about the accompanying cardiac functional/structural changes. In a total of 4,795 patients with nonvalvular AF, increases in the left ventricular mass index (LVMI) and prevalence of left ventricular hypertrophy (LVH) as well as LAVI and *E*/*E*′ were observed with elevating CHA_2_DS_2_-VASc scores (*p* < 0.05 for LVMI and LVH and *p* < 0.001 for LAVI and *E*/*E*′). LVH (hazard ratio (HR), 3.609; confidence interval (CI), 2.426–5.369; *p* < 0.001) and *E*/*E*′ (HR, 1.087; CI, 1.054–1.121; *p* < 0.001) were independent risk factors for a CHA_2_DS_2_-VASc score of 2 or higher. The authors stated that higher CHA_2_DS_2_-VASc scores are associated with impaired diastolic function, reflecting high left atrial pressure and increased risk of thromboembolism [31]. Perhaps, lowering the left ventricular end-diastolic pressure should be a therapeutic target for HFpEF patients with high CHA_2_DS_2_-VASc scores to decrease the prevalence of thromboembolic events in this group of patients [32]. The increased LAVI in the aspect of ESUS stroke may also be important as a predictor of paroxysmal AF as a true cause of stroke. Detecting AF after ischemic stroke is challenging because of its paroxysmal nature and often silent, asymptomatic course, as was confirmed in studies with implantable devices. Baturova et al. reported that left atrial dilatation assessed by LAVI independently predicted AF after stroke in patients without prior AF history, while the other clinical or ECG markers were not predictive for AF detection early after ischemic stroke. The authors suggest that initially, there is development of subtle structural changes predictive for future AF seen in echocardiography (for example, increased LAVI) [33]. The level of NT-proBNP may participate in pathogenesis and pathophysiology of ischemic stroke. The efforts to find a correlation between the NT-proBNP concentration and stroke topography, size, or gravity of neurological deficit do not give clear-cut results. The same concerns the prognostic value of BNP concentration during ischemic stroke. Despite conflicting reports, it is worth continuing this research, because among other things, there is a connection between cardiac insufficiency and prognosis in acute cerebrovascular incidents [34, 35]. In our study, ESUS patients had lower mean early diastolic (*E*′) and systolic (*S*′) mitral annular velocities and a higher *E*/*E*′ ratio as well as longer IVRT compared to the control group, which confirms the connection between indices of diastolic heart failure and ESUS stroke. Even slightly increased values of LAVI (cutoff point 24 mL/m^2^) and NT-proBNP (cutoff point 99 pg/mL) were predictors of this type of stroke [11]. Further investigation is necessary to attribute the additive values of echocardiographic parameters for stroke prediction and the effect of left ventricular end-diastolic pressure (LVEDP) control on the reduction of ischemic stroke events. LAVI may be a new noninvasive tool to identify patients after stroke who would benefit the most from continuous screening for AF.

### 5.3. Arterial Stiffness and the Risk of ESUS

Arterial stiffness has been regarded as a reliable marker of arterial structural and functional alterations after abundant experimental and clinical studies. Vascular structure, vascular function, and BP are the three major components that are involved in arterial stiffness [36]. Factors such as inflammation, oxide stress, the renin-angiotensin-aldosterone system (RAAS), and genetic factors that influence the vascular function in the short term or the structure in the long term can induce arterial stiffness [37]. Stiffening of the cervical elastic arteries may lead to cerebrovascular disease via multiple mechanisms. Increased stiffness of the carotid artery leads to a higher pulsatile pressure and flow load on the brain, which can penetrate distally into the cerebral microcirculation, causing cerebral ischemia and hemorrhage. The increased stiffness of elastic arteries may also cause excessive blood pressure variability, which may further sensitize the brain to the harmful effects of impaired microvascular vasoreactivity [38]. The increased pulsatile load may induce a hypertrophic remodeling response of small cerebral arteries, which initially limit the penetration of the pulsatile load into the microcirculatory system by raising vascular resistance, leading to impaired vasoreactivity, hypoperfusion, and chronic ischemia [39]. One of the causes of ischemic stroke is chronic atherosclerosis. The atherosclerotic state might be reflected by increased arterial stiffness, whereby the aortic pressure is augmented, resulting in increased arterial wall stress and left ventricular afterload [40]. Arterial stiffness provides important information regarding the progression of atherosclerosis and can be measured noninvasively [41, 42]. Increased arterial stiffness causes vessel damage and is independently associated with deep or infratentorial cerebral microbleeds [43, 44]. After an average 7.9 years of follow-up of middle-aged patients with essential hypertension, Laurent et al. found that a 1-SD elevation (4 cm/s) in PWV was associated with a 72% higher risk of fatal stroke. High PWV remained significantly predictive of stroke death after adjustment for classical cardiovascular risk factors. Other researchers assessed its predictive value in the elderly and general population. Byun et al. reported that increased arterial stiffness assessed based on higher values of AIx75 in acute lacunar infarction may be related to the pathogenesis of lacunar infarction [45]. Data from two recent meta-analyses suggest that the assessment of aortic or carotid stiffness could improve the prediction of stroke beyond other conventional risk factors. In addition, aortic stiffness could predict the prognosis of ischemic stroke [46]. Larger studies that evaluate the relationship between vascular stiffness and each subtype of stroke are imperative to help clarify the direct interaction in pathogenesis and provide specific insights into efficient stroke prevention. In our study, the parameters of arterial stiffness—augmentation pressure (AP), augmentation index (AIx), and augmentation index adjusted to a heart rate of 75 bpm (AIx75)—were significantly higher in ESUS patients compared to controls. The risk for recurrent stroke events remains high (the 5-year rate for recurrent stroke is 26% and at 10 years is nearly 40%), so it is important to determine whether the arterial stiffness remains elevated in the observation after stroke [42].

## Figures and Tables

**Table 1:** Basic characteristics of patients in both groups.

Parameter	Patients with ESUS (*n* = 65)	Controls (*n* = 36)	*p*
Gender (male) (%)	42	61	0.059
Median age (years)	54 (IQR 47-58)	53 (47-58)	0,89
SP (mmHg)	136 ± 18.7	128 ± 19.7	0.05
DP (mmHg)	82 ± 9.9	81, 8 ± 9.0	0.72
BMI (kg/m^2^)	26.1 (22.4-28.7)	25 (21.8-28.1)	0.49
Hypertension (%)	52	43	0.36
CAD (%)	9	9	0.76
Diabetes	8	0	0.23
Smoking (%)	38	13	0.02
ASA (%)	23	9	0.12
Statin (%)	23	17	0.66
Beta blocker (%)	28	12	0.13
ACE (%)	37	24	0.2
CCB (%)	20	9	0.27
Insulin (%)	8	0	0.23
Diuretic (%)	20	15	0.75

ASA: acetylsalicylic acid; ACE: angiotensin-converting enzyme; BMI: body mass index; CAD: coronary artery disease; CCB: calcium-channel blocker; DP: diastolic pressure; SP: systolic pressure.

**Table 2:** Basic characteristics of patients in both groups—evaluation of biochemical parameters in groups.

Parameter	Patients with ESUS (median with IQR)	Controls (median with IQR)	*p*
K^+^ (mmol/L)	4.08 (±0.35)	4.21 (±0.28)	0.058
Creatinine (*μ*m/L)	75.0 (64.0-89.0)	68.0 (62.0-78.0)	0.21
GFR (mL/min/1.73 m^3^)	75 (64-89)	68 (62-78)	0.002
NT-proBNP (pg/mL)	391 (107.9-1249.22)	109 (46.22-236.90)	0.0003
Total cholesterol (mmol/L)	4.9 ± 1.4	5.1 ± 1.17	0.057
LDL cholesterol (mmol/L)	2.83 (2.07-4.0)	3.02 (2.66-3.67)	0.37
HDL cholesterol (mmol/L)	1.19 (0.95-1.46)	1.37 (1.19-1.6)	0.02
Triglycerides (mmol/L)	1.58 (1.11-2.0)	1.33 (0.86-1.7)	0.16
APTT (s)	28.1 (25.9-31.3)	28.7 (26.5-31.7)	0.42
PT (s)	12.0 (11.5-12.6)	11.9 (11.7-12.4)	0.90
ADMA (*μ*mol/L)	0.44 (0.39-0.55)	0.36 (0.32-0.40)	0.0002
IL-6 (pg/mL)	2.6 (0.8-8.1)	0.7 (0.4- 1.2)	0.002

ADMA: asymmetric dimethylarginine; APTT: activated partial thromboplastin time; HDL: high-density lipoprotein; IL-6: interleukin 6; LDL: low-density lipoprotein; K^+^: potassium; NT-proBNP: N-terminal probrain natriuretic peptide; GFR: glomerular filtration rate; PT: prothrombin time.

**Table 3:** Evaluation of selected echocardiographic parameters in both groups.

Parameter	Patients with ESUS	Controls	*p*
*E*	69 ± 17	76 ± 17	0.09
*A*	79 ± 19	64 ± 17	0.001
*E*′ (cm/s)	8.6 (7.1-10.3)^∗^	12.5 (9.6-14)^∗^	0.0008
*S*′ (cm/s)	7.0 ± 1.0	8.0 ± 1.0	0.03
*A*′ (cm/s)	15.54 ± 4.95	16.50 ± 3.86	0.39
*E*/*E*′ (cm/s)	7.6 (6.1-8.9)^∗^	6.0 (5.3-6.9)^∗^	0.0002
LVMI (g/m^2^)	112.0 (90.0-125.5)^∗^	89.5 (77.0-101.0)^∗^	0.0004
LA (mm)	36.0 (33.0-41.0)^∗^	35.0 (32.0-38)^∗^	0.07
LAVI (mL/m^2^)	27.0 ± 11	21.0 ± 5	0.01
LVEF (%)	60 (55-64)	63 (60-66)	0.009
IVRT (m/s)	113.0 ± 23	97 ± 30	0.001
TAPSE (mm)	24.0 (21-27)^∗^	25.0 (22.0-28.0)^∗^	0.34
PL (%)	25	16	0.04

For parameters with nonnormal distribution median values, lower and higher values are given. For parameters with normal distribution mean, values ± standard deviation (SD) are given; *A*′: late mitral annular motion; *A*: late diastolic filling velocity; *E*/*E*′: ratio of peak velocity of early diastolic transmitral flow to peak velocity of early diastolic mitral annular motion as determined by pulsed wave Doppler; *E*: early diastolic filling velocity; *E*′: early diastolic mitral annular velocity; HF: high frequency; IVRT: isovolumic relaxation time; LA: left atrium; LF: low frequency; LAVI: left atrial volume index; LV: left ventricle; LVEF: left ventricular ejection fraction; LVMI: left ventricular mass index; *S*′: systolic mitral annular velocity; PL: pericardial liquid; TAPSE: tricuspid annular plane systolic excursion; ^∗^median.

**Table 4:** Evaluation of hemodynamic parameters using the SphygmoCor system in both groups.

Parameter	Patients with ESUS	Controls	*p*
AP (mmHg)	11.0 (7-18)^∗^	6.0 (3.0-13.0)^∗^	0.0018
AIx (%)	27 ± 13	22 ± 13	0.03
AIx75 (%)	25 ± 11	18 ± 12	0.009
PWV (m/s)	7.2 (6.1-8.4)^∗^	7,4 (6,2-9,4)^∗^	0.24
HRV index	6.6 (4.7-9)^∗^	8.7 (5.9-12.1)^∗^	0.006
DP aortic (mmHg)	83.0 (78.0-90.0)^∗^	81.0 (76.0-90.0)^∗^	0.59
SP aortic (mmHg)	121.28 ± 17, 72	124.64 ± 18.27	0.42

AP: augmentation pressure; AIx: augmentation index; AIx75: adjusted augmentation index at a heart rate of 75 beats per minute; DP: diastolic pressure; HRV index: heart rate velocity; SP: systolic pressure; PWV: pulse wave velocity.

**Table 5:** Univariate logistic regression analysis of the parameters in which the univariate analysis using Mann-Whitney *U* test, Student's *t*-test, or chi^2^ test differs significantly between ESUS and Control groups.

Parameter	OR	95% CI	*p*
SP (mmHg)	1,023	0,99-1,05	0,057
Smoking	4,375	1,37-13,96	0,013
Cholesterol HDL (mmol/L)	0,248	0,07-0,09	0,028
GFR (mL/min/1.73 m^3^)	0,953	0,93-0,98	0,002
ADMA (*μ*mol/L)	9042	27,5-29,6	0,002
IL-6 (pg/mL)	1,859	1,06-3,26	0,031
NT-proBNP (pg/mL)	1,002	1,001-1,003	0,004
LAVI (mL/m^2^)	1,064	1,01-1,21	0,021
LVEF (%)	0,914	0,85-0,99	0,019
*A*	1,050	1,02-1,08	0,001
IVRT (m/s)	1,037	1,02-1,06	0,001
*S*′ (cm/s)	0,763	0,59-0,99	0,044
*E*′ (cm/s)	0,087	0,77-0,99	0,027
*E*/*E*′ (cm/s)	1,465	1,14-1,88	0,003
LVMI (g/m^2^)	1,034	1,01-1,06	0,002
PL (%)	5,277	1,13-24,57	0,034
HRV index	0,995	0,96-1,03	0,784
AP (mmHg)	1,100	1,03-1,18	0,005
AIx (%)	1,035	1,01-1,07	0,038
AIx75 (%)	1,048	1,01-1,09	0,014

SP: systolic pressure; HDL: high-density lipoprotein; GFR: glomerular filtration rate; ADMA: asymmetric dimethylarginine; IL-6: interleukin 6; NT-proBNP: N-terminal probrain natriuretic peptide; LAVI: left atrial volume index; LVEF: left ventricular ejection fraction; *A*: late diastolic filling velocity; IVRT: isovolumic relaxation time; *S*′: systolic mitral annular velocity; *E*′: early diastolic mitral annular velocity; *E*/*E*′: ratio of peak velocity of early diastolic transmitral flow to peak velocity of early diastolic mitral annular motion as determined by pulsed wave Doppler; LVMI: left ventricular mass index; PL: pericardial liquid; HRV index: heart rate velocity; AP: augmentation pressure; AIx: augmentation index; AIx75: adjusted augmentation index at a heart rate of 75 beats per minute.

**Table 6:** Multivariate analysis—stepwise logistic regression.

Variable	OR	95% CI for OR		*p* value
		Lower limit	Upper limit	
IVRT (ms)	1.045	1.009	1.982	0.01
NT-proBNP (pg/mL)	1.003	1.001	1.005	0.007
LAVI (mL/m^2^)	1.3	1.099	1.537	0.002
AIx75	1.095	1.004	1.194	0.04

AIx75: adjusted augmentation index at a heart rate of 75 beats per minute; IVRT: isovolumic relaxation time; LAVI: left atrial volume index; NT-proBNP: N-terminal probrain natriuretic peptide.

## Data Availability

All data are available from the authors upon reasonable request.

## Additional Points

*Limitations*.The study involved a relatively small number of patients and the findings need to be confirmed in a larger population. Not all patients underwent transesophageal echocardiography (TEE) during the hospital stay. TEE is not included in the standardized assessment required to diagnose ESUS, but it may change patient management in a proportion of patients. The limitations of PWV measurement should be mentioned here also. It was difficult to accurately record the femoral pressure wave in participants with acute stroke, and we assessed radial-carotid PWV, which may be a reason why we did not find a significant difference in this parameter between groups. This acute increase in arterial stiffness may be caused by a complex interaction of multiple factors, including the occurrence of inflammatory processes and oxidative stress and the presence of endothelial dysfunction [42].
